# Pressurized intraperitoneal aerosol chemotherapy in advanced gastric cancer with peritoneal metastases: a comprehensive meta-analysis of feasibility, efficacy, and safety

**DOI:** 10.1093/gastro/goaf040

**Published:** 2025-06-15

**Authors:** Ruijian Chen, Zifeng Yang, Renjie Li, Yuesheng Yang, Jiabin Zheng, Junjiang Wang, Yong Li

**Affiliations:** Shantou University Medical College, Shantou, Guangdong, P. R. China; Department of Gastrointestinal Surgery, Guangdong Provincial People’s Hospital (Guangdong Academy of Medical Sciences), Southern Medical University, Guangzhou, Guangdong, P. R. China; Department of General Surgery, Guangdong Provincial People’s Hospital (Guangdong Academy of Medical Sciences), Southern Medical University, Guangzhou, Guangdong, P. R. China; Department of Gastrointestinal Surgery, Guangdong Provincial People’s Hospital (Guangdong Academy of Medical Sciences), Southern Medical University, Guangzhou, Guangdong, P. R. China; Department of General Surgery, Guangdong Provincial People’s Hospital (Guangdong Academy of Medical Sciences), Southern Medical University, Guangzhou, Guangdong, P. R. China; Department of Gastrointestinal Surgery, Guangdong Provincial People’s Hospital (Guangdong Academy of Medical Sciences), Southern Medical University, Guangzhou, Guangdong, P. R. China; Department of General Surgery, Guangdong Provincial People’s Hospital (Guangdong Academy of Medical Sciences), Southern Medical University, Guangzhou, Guangdong, P. R. China; Shantou University Medical College, Shantou, Guangdong, P. R. China; Department of Gastrointestinal Surgery, Guangdong Provincial People’s Hospital (Guangdong Academy of Medical Sciences), Southern Medical University, Guangzhou, Guangdong, P. R. China; Department of General Surgery, Guangdong Provincial People’s Hospital (Guangdong Academy of Medical Sciences), Southern Medical University, Guangzhou, Guangdong, P. R. China; Department of Gastrointestinal Surgery, Guangdong Provincial People’s Hospital (Guangdong Academy of Medical Sciences), Southern Medical University, Guangzhou, Guangdong, P. R. China; Department of General Surgery, Guangdong Provincial People’s Hospital (Guangdong Academy of Medical Sciences), Southern Medical University, Guangzhou, Guangdong, P. R. China; Department of Gastrointestinal Surgery, Guangdong Provincial People’s Hospital (Guangdong Academy of Medical Sciences), Southern Medical University, Guangzhou, Guangdong, P. R. China; Department of General Surgery, Guangdong Provincial People’s Hospital (Guangdong Academy of Medical Sciences), Southern Medical University, Guangzhou, Guangdong, P. R. China; Department of Gastrointestinal Surgery, Guangdong Provincial People’s Hospital (Guangdong Academy of Medical Sciences), Southern Medical University, Guangzhou, Guangdong, P. R. China; Department of General Surgery, Guangdong Provincial People’s Hospital (Guangdong Academy of Medical Sciences), Southern Medical University, Guangzhou, Guangdong, P. R. China

**Keywords:** pressurized intraperitoneal aerosol chemotherapy, gastric cancer, peritoneal metastases, efficacy, feasibility, safety

## Abstract

Pressurized intraperitoneal aerosol chemotherapy (PIPAC) has emerged as a promising therapeutic approach for treating advanced gastric cancer with peritoneal metastases. Herein, we conducted this meta-analysis to evaluate the feasibility, efficacy, and safety of PIPAC in this patient population. The literature between January 2011 and February 2024 was comprehensively searched on the following databases: PubMed, Embase, Web of Science, and the Cochrane Library. The search, guided by the Population-Intervention-Comparison-Outcome (PICO) framework, focused on studies reporting on the feasibility, efficacy, and safety of PIPAC. Data were pooled by using log transformation (PLN) or Freeman-Tukey double arcsine transformation. Of the 451 initially identified studies, 18 were included in the meta-analysis, comprising 671 patients who underwent 1,357 PIPAC procedures. Our data analysis indicated that 32.6% of the patients (95% confidence interval [CI], 23.5%–42.3%) completed three or more PIPAC procedures. Conversely, 2.3% of patients (95% CI, 0.6%–5%) either did not have access to or could not undergo PIPAC. The average rate of histological response across the included studies was 66.3% (95% CI, 59.1%–73.1%). Pooled results showed that 13.1% of patients (95% CI, 7.0%–20.7%) had reduced ascites after PIPAC, and 7.8% (95% CI, 4.8%–11.4%) became resectable. Adverse events were reported in 17.1% of patients (95% CI, 5.3%–33.4%), with 3.6% (95% CI, 1.4%–6.6%) experiencing severe adverse events (grade 3–5, Common Terminology Criteria for Adverse Events [CTCAE]). The pooled mortality related to PIPAC was 0.1% (95% CI, 0%–0.5%). The pooled proportions for 6-month, 1-year, and 2-year overall survival rates were 82.4% (95% CI, 69.2%–92.8%), 54.0% (95% CI, 45.7%–62.3%), and 20.0% (95% CI, 11.3%–30.3%), respectively. The average median overall survival was 11.7 months (95% CI, 9.3–14.0 months). Our study suggests that most patients can benefit from PIPAC treatment, such as improved quality of life and significantly longer median overall survival. Patients who received first-line chemotherapy prior to PIPAC and concomitant systemic chemotherapy during PIPAC treatment, and who underwent the PIPAC procedure on more than three occasions, exhibited a more favorable survival prognosis.

## Introduction

Gastric cancer is the fifth most prevalent cancer globally and ranks third among the leading causes of cancer-related mortality, according to the 2020 Global Cancer Statistics [1]. Peritoneal metastasis is a common complication in advanced gastric cancer, affecting 10%–40% of patients at initial diagnosis. This condition significantly impacts disease progression and recurrence, resulting in a median survival of only 3–7 months for patients with peritoneal metastasis [2–4].

Systemic chemotherapy remains the standard treatment of advanced gastric cancer with peritoneal metastasis. However, its efficacy is often limited by inadequate drug penetration into the peritoneum due to factors such as poor blood flow and the plasma-peritoneal barrier. Furthermore, prolonged systemic chemotherapy can lead to drug resistance and significant toxicity, further reducing its effectiveness [5–8]. Recently, cytoreductive surgery (CRS) combined with hyperthermic intraperitoneal chemotherapy (HIPEC) has emerged as an alternative treatment strategy. CRS aims to reduce tumor burden and increase sensitivity to chemotherapy, while HIPEC provides localized high-dose chemotherapy. Although this approach shows promise, it is highly selective for certain tumor types and may be less effective for gastric cancer with peritoneal metastasis. Moreover, it faces challenges related to pharmacokinetics, such as limited tumor penetration and incomplete serosal surface coverage [9–15].

In 2011, pressurized intraperitoneal aerosol chemotherapy (PIPAC) was introduced as a novel method for intraperitoneal drug delivery [16]. PIPAC has been noted for its ease of implementation, extensive drug distribution, and significant depth of drug penetration. It offers favorable pharmacological characteristics, including high local drug concentrations and minimal systemic exposure [17–19]. Despite these advantages, PIPAC is a relatively new technique, and robust large-scale prospective comparative studies are lacking. Current knowledge about dosing regimens, pressure settings, exposure durations, and timing intervals for PIPAC is largely based on empirical evidence [20, 21].

Herein, we designed this meta-analysis to systematically evaluate the feasibility, efficacy, and safety of PIPAC for treating advanced gastric cancer with peritoneal metastasis.

## Methods

### Search strategy

This systematic review and meta-analysis adhered to the Preferred Reporting Items for Systematic Reviews and Meta-Analyses (PRISMA) guidelines and the Assessing the Methodological Quality of Systematic Reviews (AMSTAR) criteria. The review was preregistered in the International Prospective Register of Systematic Reviews (PROSPERO) under registration number CRD42023413651 prior to initiating the study.

To identify relevant studies, a comprehensive search was conducted across multiple databases, including PubMed, Embase, Web of Science, and the Cochrane Library. Given that PIPAC was first reported in 2011, our search covered studies published between January 2011 and February 2024.

The review followed the Population-Intervention-Comparison-Outcome (PICO) model. The population (P) included patients with a confirmed histological diagnosis of gastric cancer with peritoneal metastasis. The intervention (I) was PIPAC. The comparison (C) was not applicable as the meta-analysis was single-arm. The outcomes (O) focused on efficacy (overall survival and histological responses) and safety (surgical complications and PIPAC-related adverse events). Search terms included “peritoneal carcinosis OR peritoneal metastases OR peritoneal carcinomatosis” AND “(gastric OR stomach) AND (Neoplasm* OR Tumor* OR Cancer* OR Carcinoma* OR carcinosis*)” AND “pressurized intraperitoneal aerosol chemotherapy OR PIPAC.” Additionally, the reference lists of retrieved articles were reviewed to identify potentially relevant studies.

### Inclusion and exclusion criteria

The inclusion criteria were as follows: (1) prospective or clinical studies; (2) articles investigating PIPAC in gastric cancer with peritoneal metastasis; and (3) studies reporting on efficacy (overall survival and histological response) and safety (surgical complications and PIPAC-related adverse events) of PIPAC. The exclusion criteria were as follows: (1) article type: review, meta-analysis, letters, conference report, case report, and animal studies; (2) articles unrelated to purpose or outcome; (3) articles without available data that can be extracted; and (4) duplicate publications.

### Data extraction

All articles were imported into Endnote, a reference management software, to remove duplicates. The remaining articles were then screened for relevance based on predefined inclusion and exclusion criteria. Following an initial screening of titles and abstracts, two independent reviewers conducted a full-text assessment and reviewed the reference lists of relevant publications to make final inclusion decisions. Any discrepancies were resolved through discussion, and a third reviewer was consulted when necessary to reach a consensus.

From the eligible publications, the following information was extracted: (1) study characteristics, including the first author’s name, year of publication, country of study, and study design; (2) study population details, such as total number of patients, median follow-up duration, age, and sex distribution; (3) intervention specifics, including the intraperitoneal regimen, dosage, number of PIPAC cycles administered, and any prior systemic treatments; and (4) outcomes, including PIPAC-related adverse events classified according to the Common Terminology Criteria for Adverse Events version 4.0 (CTCAE v4.0), median overall survival, histological response rate, and survival rates at 6 months, 1 year, and 2 years.

Histological response rate was defined as the proportion of patients with a complete or major histological response, as determined by the Peritoneal Regression Grading Score (PRGS) after the second PIPAC-Ox cycle. PRGS, calculated as the number of patients that showed any histological response to PIPAC treatment on the total number of patients evaluated for this outcome in each study [22], was defined as follows: 1 (complete regression, absence of tumor cells), 2 (major regression, few residual tumor cells), 3 (minor regression, predominance of residual tumor cells), and 4 (no response, presence of tumor cells with no regressive features). Median survival times and survival rates were extracted using OriginPro software based on published survival curves.

### Quality assessment

The risk of bias for each study was evaluated by using the Newcastle–Ottawa Scale (NOS), which includes nine indicators related to selection, comparability, and outcomes. The NOS scores range from 0 (lowest quality) to 9 (highest quality).

Two authors independently conducted the risk of bias and methodological quality assessments. Any discrepancies were resolved through discussion, and a third author was consulted if a consensus could not be reached.

### PIPAC procedure

All PIPAC procedures were performed laparoscopically by using two standard balloon trocars. Briefly, the procedure began by establishing pneumoperitoneum through the infusion of normothermic carbon dioxide, maintaining an intra-abdominal pressure of 12 mmHg. A nebulizer connected to a high-pressure syringe was then used to atomize chemotherapy drugs into an aerosol form, which was introduced into the peritoneal cavity for 30 minutes. After the exposure period, the aerosol was extracted by using a closed inhalation system. The chemotherapy drugs administered typically included doxorubicin (1.5 mg/m^2^) in 0.9% sodium chloride (50 mL) and cisplatin (7.5 mg/m^2^) in 0.9% sodium chloride (150 mL). PIPAC treatments were performed every 6 weeks ± 2 weeks, with a minimum of three cycles recommended. Concurrent systemic therapy was possible but generally advised against for 2 weeks before and 1 week after PIPAC treatment.

### Statistical analysis

We employed standard meta-analysis techniques to evaluate the outcomes using both fixed and random effects models. Statistical heterogeneity was assessed with the *I*^2^ inconsistency statistic. When significant heterogeneity was detected (*I*^2^ > 50% or *P *< 0.05), we reported results from the random-effects model. Our meta-analysis focused on several key outcomes related to PIPAC, including adverse events (graded 3 and above according to the Common Terminology Criteria for Adverse Events), median overall survival, histological response rates, 1-year overall survival rates, peritoneal cancer index (PCI) at the first PIPAC procedure, mean operating time, and demographic characteristics of the study populations.

The analysis was conducted by using the “metaprop” function within the R statistical software (version 4.2.2; https://www.r-project.org). To address challenges associated with extreme rates (close to 0% or 100%), we utilized the exact binomial method for pooling proportions and applied the Freeman-Tukey double arcsine transformation (PFT). We calculated 95% confidence intervals (CIs) using the Clopper-Pearson exact method. When data did not conform to normality, even after the Freeman-Tukey transformation, log transformation (PLN) was applied.

We performed subgroup analyses to explore potential sources of heterogeneity, considering variables such as PCI, age, sex, prior first-line systemic chemotherapy, concurrent systemic chemotherapy, and the number of PIPAC procedures performed (more than three). Statistical significance was set at *P* values <0.05.

## Results

### Study selection

**Figure 1.**
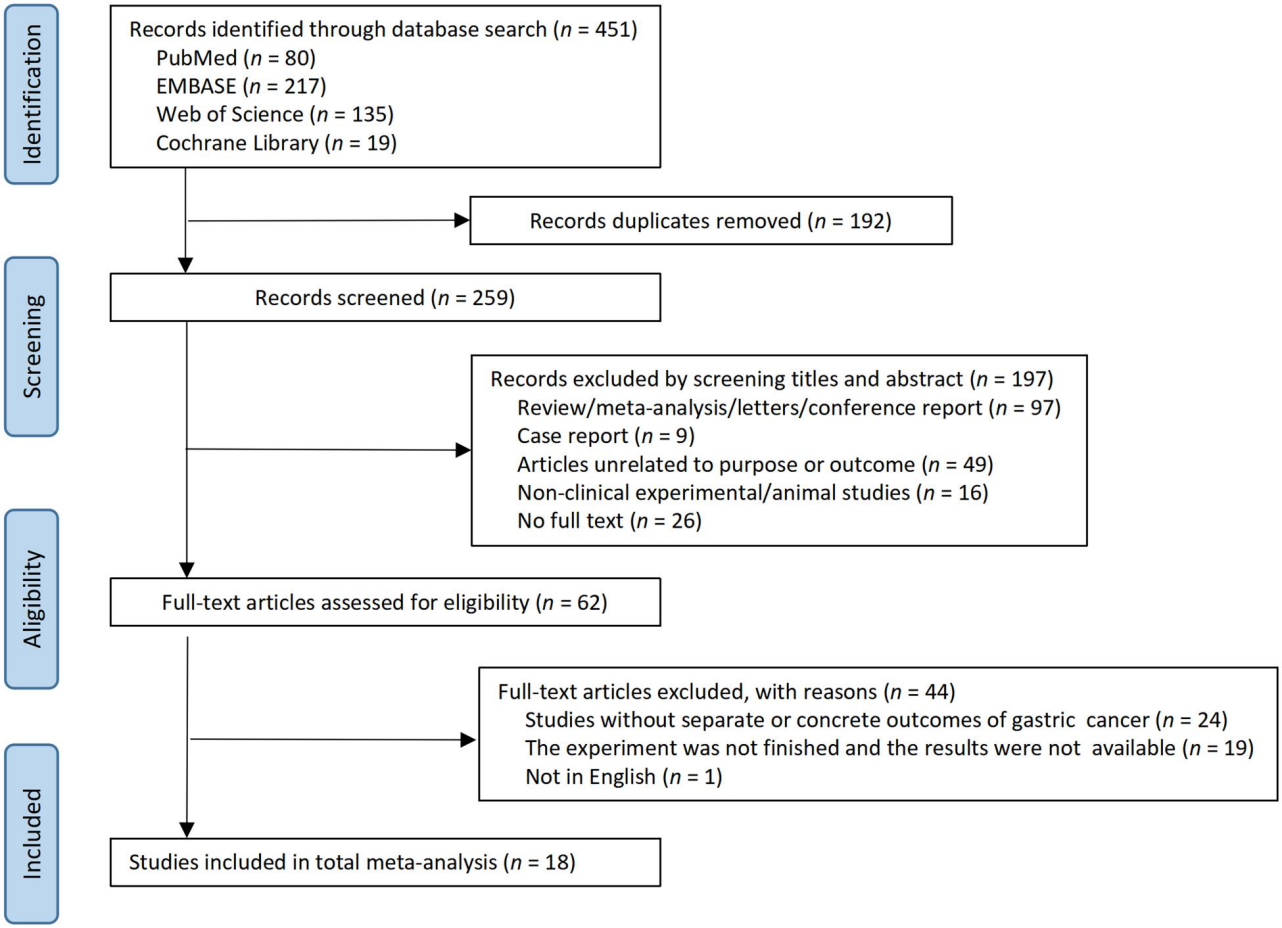
The flow chart of data screening in the study cohort

Figure 1 illustrates the study selection process. Initially, we identified a total of 451 studies across four databases: 80 from PubMed, 217 from EMBASE, 135 from Web of Science, and 19 from the Cochrane Library (Supplementary Table S1). After excluding 192 duplicate articles, we screened the titles and abstracts of the remaining 259 articles, which led to a full-text review of 62 articles, from which 18 [15, 23–39] were deemed eligible for inclusion in the meta-analysis and systematic review. The quality of these studies was assessed by using NOS. Supplementary Table S2 details the quality assessment scores, ranging from a minimum of 4 to a maximum of 9.

### Patient characteristics

Table 1 summarizes the data extracted from the 18 included studies. Of these studies, 16 were conducted in Europe, and 2 were in Asia. Among them, only one was a multi-center study; the remaining were single-center studies, comprising 11 retrospective studies, 4 prospective studies, and 2 Phase II clinical trials. The patient populations in these studies included individuals with advanced gastric cancer and peritoneal metastasis who received PIPAC treatment. The regimen included cisplatin at a dose of 7.5 mg/m^2^ and doxorubicin at a dose of 1.5 mg/m^2^.

**Table 1.** Clinical information and patient characteristics from the eligible studies

Authors	Year	Country	Study period	Study type	Center	Number of patients	Age (years), mean ± SD	Total number of PIPAC	Outcome parameters
Reymond *et al.*	2014	Germany	2011.12-2012.12	Retrospective cohort	Single	7	60.6 ± 16.4	16	BCD
Nadiradze *et al.*	2015	Germany	2011.7-2013.11	Retrospective cohort	Single	24	56.0 ± 13.0	60	ABCDGHI
Khomyakov *et al.*	2016	Russia	2013.8-2016.6	Phase II	Single	31	52.0 ± 11.0	56	BCDGHIJ
Gockel *et al.*	2018	Germany	2015.12-2018.2	Prospective	Single	24	57.7 ± 8.0	46	ABCDFGHI
Khomyakov *et al.*	2019	Russia	–	Retrospective cohort	Single	94	53.0	214	BGI
Struller *et al.*	2019	Germany	2013.11-2017.6	Phase II	Single	25	55.1 ± 13.0	43	ABCDEG
Alyami *et al.*	2020	France	–	Retrospective cross-sectional	Single	42	51.8 ± 9.7	163	CDEFGHIJ
Di Giorgio *et al.*	2020	Italy	2017.9-2019.9	Retrospective cohort	Single	28	50 ± 14.1	46	ABCDEFGI
Di Giorgio *et al.*	2020	Italy	2017.9-2019.2	Retrospective cohort	Single	15	–	27	BCDFG
Ellebæk *et al.*	2020	Denmark	2015.3-2018.10	Prospective	Single	20	56.1 ± 10.4	52	BCDGHIJ
Casella *et al.*	2021	Italy	2019.2-2021.5	Retrospective cohort	Single	21	–	28	G
Feldbrügge *et al.*	2021	Germany	2017.3-2020.5	Retrospective cohort	Single	50	57.2 ± 10.0	90	ACDE
Sindayigaya *et al.*	2021	France	–	Prospective	Single	144	56.8 ± 12.6	296	ABCDEGHIJ
Horvath *et al.*	2022	Germany	2016.4-2021.9	Retrospective cohort	Single	44	50.3 ± 12.5	93	ABG
Tidadini *et al.*	2022	France	2016.7-2020.9	Retrospective cohort	Single	17	64.0 ± 11.7	42	AEGHIJ
Kryh-Jensen *et al.*	2023	Denmark	–	Retrospective cohort	Single	39	59.8 ± 11.1	–	ABG
Graversen *et al.*	2023	Denmark	2020.03-2022.06	Prospective	Multi	21	58.8 ± 15.1	21	CDHIJ
Santullo *et al.*	2023	Italy	2016.01-2021.06	Retrospective cohort	Single	25	58.5 ± 11.7	–	HIJ

**A**. Non-access; **B**. Histological response; **C**. Severe adverse events (CTCAE 3–5); **D**. PIPAC-related mortality; **E**. Became resectable after PIPAC; **F**. Ascites decreased after PIPAC; **G**. Median OS; **H**. 6-month OS; **I**. 1-year OS; **J**. 2-year OS. CTCAE = Common Terminology Criteria for Adverse Events, PIPAC = pressurized intraperitoneal aerosol chemotherapy, OS= overall survival, SD = standard deviation.

Supplementary Figure S1 presents a forest plot illustrating the basic characteristics of the patients. Data analysis of these 18 studies indicated a mean age of 55.1 ± 2.8 years, with 44.9% of the patients being male. Data for prior chemotherapy showed that 89.5% of patients had received first-line chemotherapy, 58.7% second-line, and 16.5% third-line chemotherapy before undergoing PIPAC. Concurrent systemic chemotherapy was administered to an average of 75.9% of patients. Data on the PCI at the time of the first PIPAC procedure were available from 14 studies (*n *= 589 patients) [15, 23–25, 27–33, 35–37], with an average PCI of 18.2 ± 3.5. Of these patients, 56.5% had synchronous peritoneal metastasis, and 54.1% had undergone tumor resection prior to PIPAC. The mean duration of the PIPAC procedure was 94.5 ± 13.8 minutes, and the average post-procedure hospitalization duration was 5.4 ± 3.2 days.

### Feasibility

**Figure 2.**
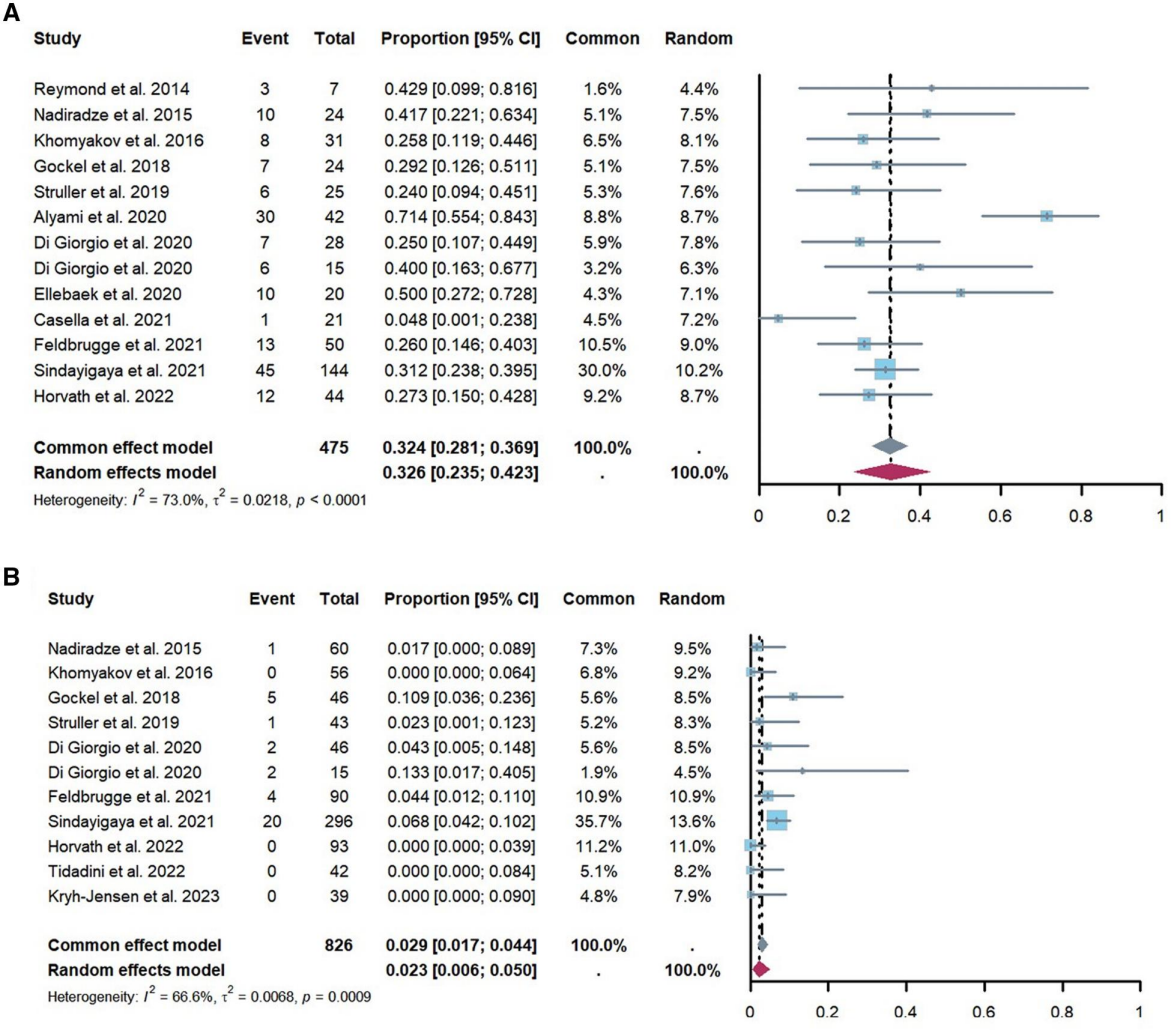
Forest plot illustrating the pooled data on feasibility. (**A**) The prevalence of PIPAC procedures performed more than three times. (**B**) The non-access rate or the inability to perform PIPAC. PIPAC = pressurized intraperitoneal aerosol chemotherapy, CI = confidence interval

This analysis included data from 18 studies (*n *= 671 patients), with a total PIPAC procedures of 1,357. Among these studies, difficulties in accessing the abdominal cavity during the initial PIPAC were reported in 35 patients, resulting in 636 successful first PIPAC procedures, and 364 patients underwent three or more PIPAC procedures. The overall proportion of patients who completed three or more PIPAC procedures was 32.6% (95% CI, 23.5%–42.3%) (Figure 2A) [15, 23–31, 33–35]. The proportion of patients who were unable to undergo PIPAC, as reported by 11 studies [15, 25, 26, 28–31, 33, 35, 36, 38], was 2.3% (95% CI, 0.6%–5%) (Figure 2B), which was mainly due to abdominal cavity adhesions and disease progression.

Significant heterogeneity was observed in the rates of non-accessibility or inability to perform PIPAC across studies (*I^2^* = 67%, *P *< 0.01). However, an analysis of variables such as the proportion of patients undergoing more than three PIPAC procedures, PCI at the time of the first PIPAC procedure, sex, age, prior first-line systemic chemotherapy, and concurrent systemic chemotherapy did not identify factors that could explain this heterogeneity (Supplementary Table S3).

### Efficacy

**Figure 3.**
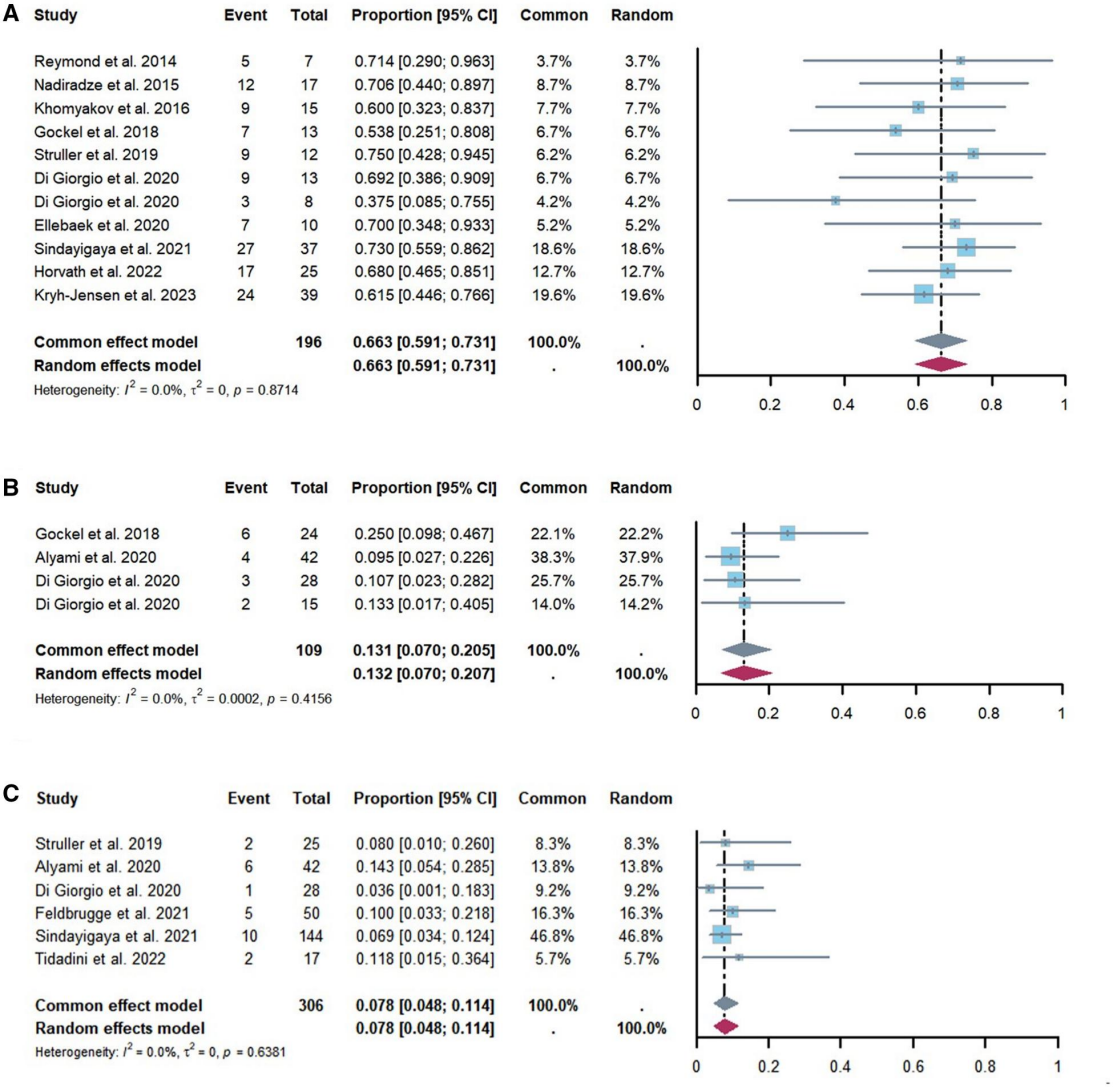
Forest plot depicting pooled data on efficacy. (**A**) The prevalence of histological responses, (**B**) the proportion of patients with reduced ascites following PIPAC, and (**C**) the proportion of patients who became resectable after PIPAC. PIPAC = pressurized intraperitoneal aerosol chemotherapy, CI = confidence interval

Histological response rates were reported in 11 studies [15, 25–27, 29–31, 33–35, 38] involving 196 patients, and the pooled average histological response rate was 66.3% (95% CI, 59.1%–73.1%) (Figure 3A). Additionally, four studies [23, 25, 26, 29] reported data on the proportion of patients whose ascites decreased following PIPAC, which was 13.1% (95% CI, 7.0%–20.7%) (Figure 3B). Among 306 patients assessed in six studies [15, 23, 25, 28, 35, 36], the pooled proportion of those who became resectable after PIPAC was 7.8% (95% CI, 4.8%–11.4%) (Figure 3C). Importantly, no significant heterogeneity was observed in the results for histological response rates (*I*^2^ = 0%, *P *= 0.84), the proportion of patients with reduced ascites (*I*^2^ = 0%, *P *= 0.42), or those who became resectable after PIPAC (*I*^2^ = 0%, *P *= 0.64).

### Safety

**Figure 4.**
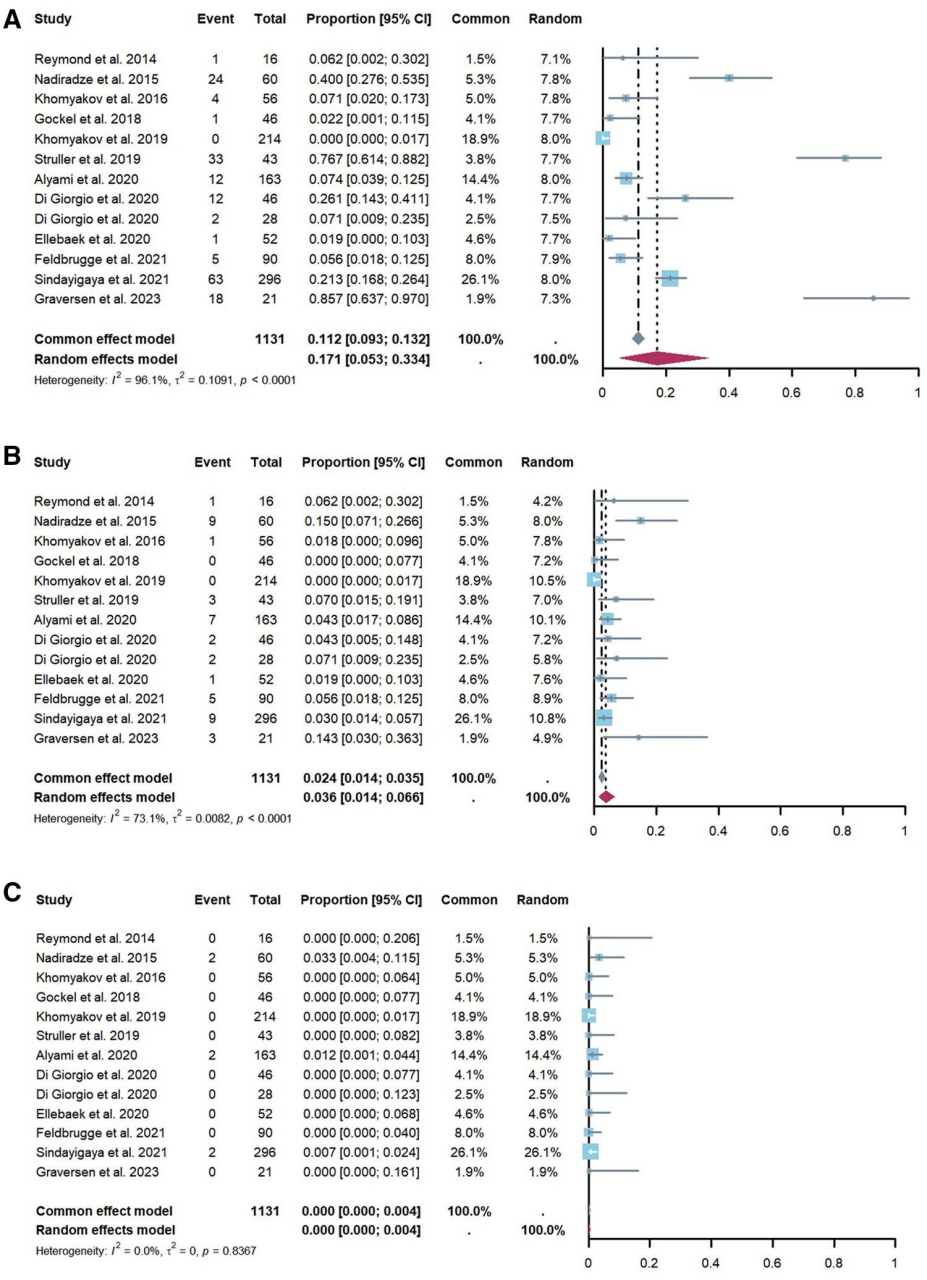
Forest plot of pooled data on safety. (**A**) The prevalence of adverse events (CTCAE grades 1–5), (**B**) the prevalence of severe adverse events (CTCAE grades 3–5), and (**C**) the prevalence of PIPAC-related mortality (CTCAE grade 5). PIPAC = pressurized intraperitoneal aerosol chemotherapy, CTCAE = Common Terminology Criteria for Adverse Events, CI = confidence interval

The safety profile of PIPAC was evaluated by assessing reported adverse events and disease-related mortality. Data on the proportion of adverse events (CTCAE grades 1–5) were obtained from 13 studies [15, 23–29, 31–35, 39] involving 1,131 PIPAC procedures. The overall proportion of adverse events was 17.1% (95% CI, 5.3%–33.4%) (Figure 4A), with severe adverse events (CTCAE grades 3–5) occurring in 3.6% of patients (95% CI, 1.4%–6.6%) (Figure 4B) [15, 23–29, 31–35, 39]. Significant heterogeneity was observed in the rates of severe adverse events (CTCAE 3–5) across studies (*I*^2^  = 73%, *P* < 0.01). However, factors such as the PCI at the time of the first PIPAC procedure, sex, age, previous first-line systemic chemotherapy, concomitant systemic chemotherapy, and the proportion of patients who underwent PIPAC procedures more than three times did not account for this heterogeneity (Supplementary Table S3).

Regarding PIPAC-related mortality, analysis of the evaluated studies revealed that six out of PIPAC procedures [15, 23–29, 31–35, 39] experienced severe adverse events leading to death (CTCAE grade 5), with disease progression being the primary cause. The pooled proportion of PIPAC-related mortality was 0.1% (95% CI, 0%–0.4%) (Figure 4C). No significant heterogeneity was observed in the mortality data (*I^2^* = 0%, *P *= 0.84).

### Survival analysis

**Figure 5.**
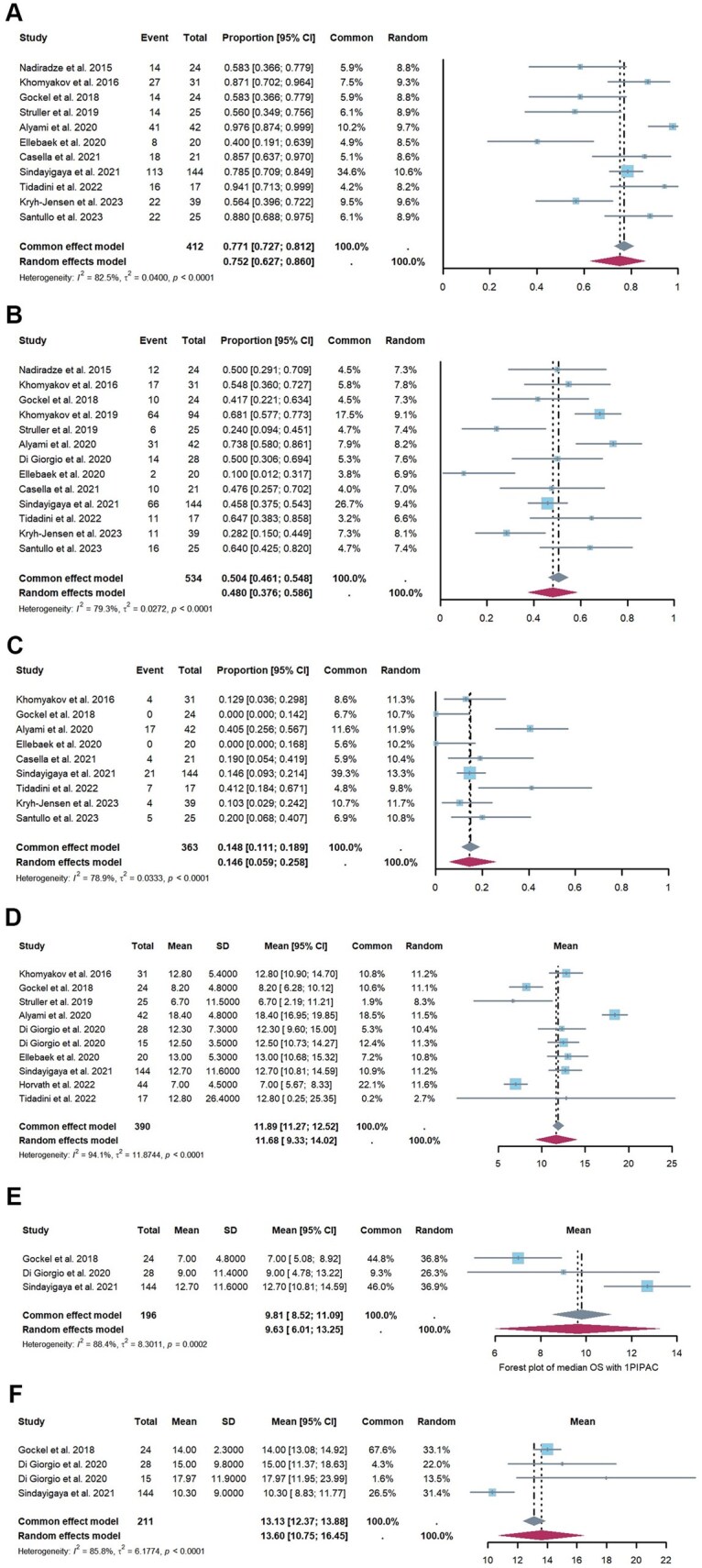
Forest plot showing the pooled prevalence of overall survival at 6 months (**A**), 1 year (**B**), and 2 years (**C**). (**D**) The median overall survival for all patients, and (**E** and **F**) the median overall survival for patients who underwent one PIPAC and more than one PIPAC, respectively. PIPAC = pressurized intraperitoneal aerosol chemotherapy, CI = confidence interval

Survival outcomes associated with PIPAC were analyzed, revealing the following pooled overall survival rates: 82.4% at 6 months (95% CI, 69.2%–92.8%), 54.0% at 1 year (95% CI, 45.7%–62.3%), and 20.0% at 2 years (95% CI, 11.3%–30.3%) (Figure 5A–C). Median overall survival data from 10 studies [15, 23, 25–27, 29–31, 35, 36] involving 390 patients indicated an average of 11.7 months (95% CI, 9.3–14.0 months) (Figure 5D). Notably, patients who underwent more than one PIPAC procedure [15, 25, 26, 29] demonstrated a significantly longer median overall survival compared to those who had only one procedure [15, 25, 29] (13.6 months vs 9.6 months) (Figure 5E and F).

In analyzing survival outcomes associated with PIPAC, we observed significant statistical heterogeneity in the overall survival rates and median overall survival times. Specifically, heterogeneity was high for the 6-month overall survival rate (*I^2^* = 84%, *P *< 0.01), the 1-year overall survival rate (*I^2^* = 66%, *P *< 0.01), and the 2-year overall survival rate (*I^2^* = 70%, *P *< 0.01). Similarly, heterogeneity was evident in median overall survival times (overall: *I^2^* = 94%, *P *< 0.01; with 1 PIPAC: *I^2^* = 88%, *P *< 0.01; with >1 PIPAC: *I^2^* = 86%, *P *< 0.01). To address this variability, we performed meta-regression and subgroup analyses focusing on the 1-year survival rate and median overall survival time (Supplementary Table S3). Our analysis of the 1-year survival rate revealed that concomitant systemic chemotherapy and previous first-line systemic chemotherapy were significant contributors to the observed heterogeneity. Higher proportions of patients receiving concomitant systemic chemotherapy during PIPAC or those with prior first-line systemic chemotherapy were associated with improved 1-year overall survival rates (65.9% vs 47.0%, *P *< 0.01; 69.0% vs 52.8%, *P *= 0.02) (Supplementary Figures S2 and S3). These findings suggest that both concomitant systemic chemotherapy and previous first-line systemic chemotherapy may be beneficial for improving the 1-year overall survival rate in patients with gastric cancer and peritoneal metastasis. Regarding median overall survival time, sex and the proportion of PIPAC procedures performed more than three times were significant sources of heterogeneity (*P *= 0.01, and *P *= 0.02). Female patients demonstrated better prognoses, as indicated by the median overall survival times in groups with less than 50% male patients compared with those with more than and equal to 50% male patients (13.0 months vs 7.4 months, *P *= 0.01) (Supplementary Figure S4). Additionally, patients with gastric cancer and peritoneal metastasis who underwent more than 30% of their PIPAC procedures more than three times had a longer median overall survival than those who underwent fewer than and equal to 30% such procedures (14.2 months vs 9.5 months, *P *= 0.02) (Supplementary Figure S5). However, other variables did not account for the remaining heterogeneity.

## Discussion

In this study, we conducted a meta-analysis and systematic review of 18 articles on PIPAC for peritoneal metastasis in gastric cancer patients. Our study suggests that most patients can benefit from PIPAC treatment, such as improved quality of life and significantly longer median overall survival. In addition, PIPAC was found to provide significant promise in terms of feasibility, effectiveness, and safety.

Peritoneal metastasis is frequently encountered in advanced gastric cancer and is associated with a poor prognosis. Traditional treatments, such as intraperitoneal chemotherapy (IPC), CRS, and HIPEC, have demonstrated limited efficacy. Compared with alternative therapeutic modalities, PIPAC offers several distinct advantages. First, PIPAC improves the delivery of chemotherapy drugs directly to the peritoneal cavity. Traditional systemic chemotherapy often encounters challenges in effectively reaching peritoneal tumors due to the poor blood supply of the peritoneum and the presence of the peritoneal-plasma barrier, which reduces the concentration of chemotherapy drugs in the peritoneal cavity while maintaining high levels in the bloodstream, resulting in diminished therapeutic efficacy and increased systemic toxicity. However, the use of PIPAC circumvents this issue by directly administering chemotherapy in aerosol form, ensuring more effective drug delivery to the peritoneal surface. Second, PIPAC achieves a more uniform distribution of chemotherapy drugs throughout the abdominal cavity. Unlike CRS + HIPEC, which delivers chemotherapy drugs in liquid form, PIPAC uses aerosolized drugs. This method facilitates a more even dispersal of the drug across the peritoneal surface, enhancing treatment effectiveness and reducing areas of inadequate drug exposure. Lastly, PIPAC allows for the adjustment of various parameters to optimize drug penetration. By modifying factors such as pressure, temperature, and electrostatic charge, PIPAC can improve the depth and concentration of chemotherapy drugs within the peritoneal tissue. This flexibility in treatment parameters helps to overcome some of the limitations associated with traditional chemotherapy administration methods.

The concept of “therapeutic pneumoperitoneum,” introduced by Reymond *et al.* [40] in 2000, led to the development of PIPAC for advanced cancer with peritoneal metastasis [41]. Since its inception, PIPAC has garnered significant global interest and is being investigated by numerous research centers worldwide.

Regarding feasibility, our findings indicate that 32.6% of patients successfully completed three or more PIPAC procedures, with Alyami *et al.* [23] reporting the highest completion rate at 71.4%. Although there is a consensus recommending more than three PIPAC cycles, many patients are unable to complete the full course. We observed that 2.3% of patients could not undergo the initial PIPAC procedure, primarily due to peritoneal adhesions. While data on the discontinuation of subsequent PIPAC cycles are limited, disease progression and clinical deterioration are known factors contributing to such outcomes [42].

A notable number of patients had previously undergone systemic chemotherapy or surgical interventions such as CRS and HIPEC before PIPAC. Our study found that 50% of patients had prior tumor resection surgery, which increased the risk of peritoneal adhesions. This finding aligns with the observations of Jansen-Winkeln *et al.* [43], who reported that prior surgical procedures can increase the likelihood of adhesion formation. Additionally, patients with a history of CRS combined with HIPEC had a significantly higher risk (odds ratio of 5.9) of inability to access the abdominal cavity, leading to failed PIPAC administration. Despite these challenges, PIPAC remains a viable option for the majority of advanced cancer patients, even though a minority may encounter difficulties due to previous treatments.

Existing studies consistently highlight the significant clinical benefits of PIPAC in managing advanced-stage cancer with peritoneal dissemination. Our research findings demonstrate that 66.3% of patients achieved histological responses and tumor remission through PIPAC treatment, as assessed by the PRGS indicator. Additionally, 51.4% of patients presented with ascites before undergoing PIPAC treatment. Malignant ascites, which often accompanies peritoneal carcinoma, is associated with a poorer prognosis and a life expectancy typically measured in weeks to months, thereby significantly impairing patients’ quality of life [44, 45]. For symptomatic patients, the reduction of intraperitoneal fluid can improve quality of life [46]. Our study found that 13.2% of patients experienced a reduction in ascites following PIPAC treatment. However, the timing of this outcome evaluation after multiple treatment cycles was not specified in the initial studies.

Furthermore, our analysis shows that PIPAC is frequently administered to patients who have exhausted other treatment options, such as CRS or other surgical interventions. Consequently, PIPAC serves as a salvage treatment for patients with advanced-stage gastric cancer. Following repeated PIPAC procedures performed with palliative intent, some patients may become eligible for secondary CRS and HIPEC. An exploratory analysis by Girshally *et al.* [47] in 2016 assessed PIPAC’s potential as a neoadjuvant treatment for patients with peritoneal dissemination who were not suitable candidates for CRS and HIPEC. Alyami *et al.* [23] reported that 14.4% of patients who were previously considered inoperable and managed palliatively with PIPAC subsequently met the criteria for secondary CRS and HIPEC. Our findings suggest that 7.8% of patients who underwent multiple PIPAC procedures achieved conversion to secondary CRS and HIPEC, which represents a promising result, indicating that repeated PIPAC treatment may reduce peritoneal dissemination and tumor burden, potentially qualifying patients for CRS, HIPEC, or even curative surgery. Thus, PIPAC may serve as an effective neoadjuvant therapy for CRS and HIPEC.

Recent research has proposed a bidirectional treatment approach combining PIPAC with systemic chemotherapy, suggesting that this combination may be feasible and effective. Pathological responses indicate potential antitumor efficacy. Several PIPAC centers have adopted this combined approach [24, 48]. However, a systematic review has not conclusively determined whether bidirectional therapy is superior to or less effective than PIPAC alone [49]. Future research could focus on prospective trials to evaluate bidirectional therapy as a potential first-line treatment of advanced-stage cancer with peritoneal dissemination.

In terms of safety, our study reports an overall adverse event incidence rate of 17.1%, ranging from 0% to 85.7%. Severe adverse events (CTCAE 3–5) occurred at a rate of 3.6%, with a range of 0% to 14.3%. The most frequently reported symptoms were abdominal pain and intestinal obstruction, with fewer patients experiencing hepatic and renal toxicity or allergic reactions. Our research indicates that PIPAC provides higher local drug availability than traditional systemic chemotherapy, allowing for a reduction in drug dosage of up to 90%, while maintaining or even increasing tumor drug concentrations. This reduction in dosage is a key factor in the decreased incidence of toxicity reactions. A clinical study assessing renal and hepatic toxicity following PIPAC found no clinically significant hepatic cytotoxicity, and kidney function remained normal [50]. This benefit is significant, as it substantially enhances patient quality of life [51].

In terms of survival, our findings show promising outcomes for patients undergoing PIPAC treatment. Survival rates following PIPAC were encouraging, with 6-month, 1-year, and 2-year survival rates of 82.4%, 54.0%, and 20.0%, respectively. The median survival time was 11.7 months, with the longest median survival reported by Alyami *et al.* [23] at 18.4 months. Given that many patients receiving palliative PIPAC treatment had already missed the opportunity for CRS and HIPEC, it is plausible that initiating PIPAC treatment earlier, shortly after a diagnosis of peritoneal metastasis, might extend the median survival beyond 11.7 months. Furthermore, our analysis indicates that patients undergoing multiple PIPAC cycles had a median overall survival of nearly 4 months longer than those receiving a single cycle. This finding highlights the potential benefits of repeated PIPAC treatments. Despite the associated complications and adverse reactions, the extension of survival by 4 months and the improvement in quality of life underscore the value of PIPAC treatment. However, we lacked survival data for patients who underwent more than three PIPAC cycles, as this information was not consistently reported in the studies reviewed, limiting our ability to fully assess the long-term survival benefits of PIPAC.

Despite the interesting findings reported in this present study, there were several limitations that should be considered. First, as previously noted, the absence of quality of life assessments limits our understanding of the broader impacts of PIPAC treatment. Future research could address this gap to provide a more comprehensive evaluation of outcomes. Second, many of the included studies had small sample sizes, which might have introduced biases and affected the generalizability of the findings. Additionally, we observed considerable heterogeneity in patient characteristics across the studies, which could further complicate the interpretation of our reported results.

In conclusion, the findings of this study indicate that patients who received first-line chemotherapy prior to PIPAC and concomitant systemic chemotherapy during PIPAC treatment, and who underwent the PIPAC procedure on more than three occasions, exhibited a more favorable survival prognosis. Thus, PIPAC was found to provide significant promise in terms of feasibility, effectiveness, and safety. However, it remains a novel treatment modality, and the current evidence largely comes from retrospective studies. As of 1 March 2024, 19 clinical trials investigating PIPAC for gastric cancer with peritoneal dissemination have been registered on ClinicalTrials.gov., representing large-scale prospective clinical trials essential for thoroughly validating the efficacy, feasibility, and safety of PIPAC. Until now, the clinical adoption of PIPAC remains investigational until further validation results are reported.

## Supplementary Material

goaf040_Supplementary_Data

## Data Availability

Data available on request from the authors.
