# Supplementary material for: Pressurized intraperitoneal aerosol chemotherapy in advanced gastric cancer with peritoneal metastases: a comprehensive meta-analysis of feasibility, efficacy, and safety
Source: Gastroenterol Rep (Oxf). 2025 Jun 15;13:goaf040. doi: 10.1093/gastro/goaf040 (PMC12167634; doi:10.1093/gastro/goaf040)
Supplement: goaf040_Supplementary_Data [file goaf040_supplementary_data.zip › 2024-231 Supplementary tables.docx]

**Supplementary Table S1.** Screening results of four databases.

| **PubMed** | | |
| --- | --- | --- |
| **Set** | **Search Item** | **Result** |
| #1 | gastric OR stomach | 464,985 |
| #2 | neoplasm* OR tumor* OR cancer* OR carcinoma* OR carcinosis* | 5,240,894 |
| #3 | peritoneal carcinosis OR peritoneal metastases OR peritoneal carcinomatosis | 38,752 |
| #4 | pressurized intraperitoneal aerosol chemotherapy OR PIPAC | 379 |
| #5 | #1 AND #2 AND #3 AND #4 | 80 |
| **Web of Science** | | |
| **Set** | **Search Item** | **Result** |
| #1 | gastric OR stomach | 743,658 |
| #2 | neoplasm* OR tumor* OR cancer* OR carcinoma* OR carcinosis* | 8,168,879 |
| #3 | peritoneal carcinosis OR peritoneal metastases OR peritoneal carcinomatosis | 23,483 |
| #4 | pressurized intraperitoneal aerosol chemotherapy OR PIPAC | 498 |
| #5 | #1 AND #2 AND #3 AND #4 | 135 |
| **Embase** | | |
| **Set** | **Search Item** | **Result** |
| #1 | gastric OR stomach | 718,820 |
| #2 | neoplasm* OR tumor* OR cancer* OR carcinoma* OR carcinosis* | 7,384,167 |
| #3 | peritoneal carcinosis OR peritoneal metastases OR peritoneal carcinomatosis | 33,996 |
| #4 | pressurized intraperitoneal aerosol chemotherapy OR PIPAC | 653 |
| #5 | #1 AND #2 AND #3 AND #4 | 217 |
| **Cochrane Library** | | |
| **Set** | **Search Item** | **Result** |
| #1 | gastric OR stomach | 41,159 |
| #2 | neoplasm* OR tumor* OR cancer* OR carcinoma* OR carcinosis* | 277,130 |
| #3 | peritoneal carcinosis OR peritoneal metastases OR peritoneal carcinomatosis | 684 |
| #4 | pressurized intraperitoneal aerosol chemotherapy OR PIPAC | 42 |
| #5 | #1 AND #2 AND #3 AND #4 | 19 |

**Supplementary Table S2.** Quality evaluation of the eligible studies with Newcastle-Ottawa scale (NOS).

| **Study** | **Selection** | | | | **Comparability** | | **Outcome** | | | **Summary score (maximum of 9 stars)** |
| --- | --- | --- | --- | --- | --- | --- | --- | --- | --- | --- |
|  | Representative-ness | Selection of  non-exposed | Ascertainment  of exposure | Outcome not  present at start | Comparability on  most important factors | Comparability on  other risk factors | Assessment  of outcome | Long enough follow-up  (median≥ l year) | Adequacy (completeness) of follow-up |  |
| Alyami et al. | * | - | * | * | * | * | * | - | - | **6** |
| Casella et al. | * | - | * | * | - | - | * | - | - | **4** |
| Di Giorgio et al. | * | - | * | * | * | * | * | - | - | **6** |
| Di Giorgio et al. | * | - | * | * | - | - | * | - | - | **4** |
| Ellebæk et al. | * | - | * | * | * | - | * | * | * | **7** |
| Feldbrügge et al. | * | - | * | * | * | * | * | - | - | **6** |
| Gockel et al. | * | - | * | * | * | - | * | - | - | **5** |
| Horvath et al. | * | - | * | * | * | * | * | - | - | **6** |
| Khomyakov et al. | * | - | * | * | * | * | * | - | * | **7** |
| Khomyakov et al. | * | - | * | * | - | - | * | - | - | **4** |
| Nadiradze et al. | * | - | * | * | * | * | * | * | * | **8** |
| Reymond et al. | * | - | * | * | - | - | * | * | * | **6** |
| Sindayigaya et al. | * | - | * | * | * | - | * | * | * | **7** |
| Struller et al. | * | - | * | * | * | * | * | - | - | **6** |
| Tidadini et al. | * | * | * | * | * | * | * | * | * | **9** |
| Kryh-Jensen et al. | * | - | * | * | - | - | * | * | * | **6** |
| Graversen et al. | * | - | * | * | * | * | * | - | - | **6** |
| Santullo et al. | * | * | * | * | - | * | * | - | * | **7** |

**Supplementary Table S3.** Subgroup analysis of non-access rate or inability, severe adverse events (CTCAE 3–5), PIPAC-related mortality (CTCAE 5), 1 year of overall survival, and median overall survival. PIPAC = Pressurized IntraPeritoneal Aerosol Chemotherapy; CTCAE = Common Terminology Criteria for Adverse Events; PCI = Peritoneal Cancer Index.

| **Subgroup** | **Number of studies included** |  | **Heterogeneity** | |  | **Effect model** |  | **Meta analysis** | | |
| --- | --- | --- | --- | --- | --- | --- | --- | --- | --- | --- |
|  |  |  | ***P*** | *I*2 |  |  |  | **Proportion** | **95% CI** | ***P*** |
| **Non-access rate or inability to perform PIPAC** |  |  |  |  |  |  |  |  |  |  |
| PCI |  |  |  |  |  |  |  |  |  |  |
| <18 | 5 |  | 0.02 | 66% |  | random |  | 0.035 | 0.007-0.079 | 0.36 |
| ≥18 | 4 |  | 0.05 | 61% |  | random |  | 0.014 | 0.000-0.050 |  |
| Age (years) |  |  |  |  |  |  |  |  |  |  |
| <56 | 4 |  | 0.02 | 44% |  | common |  | 0.006 | 0.000-0.023 | 0.19 |
| ≥56 | 6 |  | 0.15 | 62% |  | random |  | 0.033 | 0.009-0.066 |  |
| Gender (Male%) |  |  |  |  |  |  |  |  |  |  |
| <50% | 5 |  | 0.02 | 64% |  | random |  | 0.023 | 0.021-0.059 | 0.90 |
| ≥50% | 5 |  | <0.01 | 71% |  | random |  | 0.021 | 0.000-0.065 |  |
| Previous first line systemic chemotherapy (%) |  |  |  |  |  |  |  |  |  |  |
| <100% | 6 |  | 0.01 | 67% |  | random |  | 0.030 | 0.005-0.070 | 0.85 |
| 100% | 4 |  | 0.14 | 45% |  | common |  | 0.025 | 0.004-0.057 |  |
| Concomitant systemic chemotherapy (%) |  |  |  |  |  |  |  |  |  |  |
| <100% | 6 |  | <0.01 | 47% |  | random |  | 0.040 | 0.015-0.073 | 0.50 |
| 100% | 4 |  | 0.07 | 64% |  | random |  | 0.017 | 0.000-0.073 |  |
| PIPAC procedure more than 3 times (%) |  |  |  |  |  |  |  |  |  |  |
| <30% | 6 |  | <0.01 | 68% |  | random |  | 0.024 | 0.002-0.063 | 0.30 |
| ≥30% | 3 |  | 0.14 | 49% |  | common |  | 0.051 | 0.029-0.078 |  |
| Study type |  |  |  |  |  |  |  |  |  |  |
| Retrospective | 6 |  | 0.04 | 57% |  | random |  | 0.017 | 0.000-0.051 | 0.47 |
| Prospective | 4 |  | 0.02 | 71% |  | random |  | 0.040 | 0.005-0.099 |  |
| **Severe adverse events (CTCAE 3–5)** |  |  |  |  |  |  |  |  |  |  |
| PCI |  |  |  |  |  |  |  |  |  |  |
| <18 | 8 |  | <0.01 | 80% |  | random |  | 0.028 | 0.005-0.063 | 0.34 |
| ≥18 | 2 |  | 0.85 | 0% |  | common |  | 0.051 | 0.018-0.096 |  |
| Age (years) |  |  |  |  |  |  |  |  |  |  |
| <56 | 4 |  | 0.67 | 0% |  | common |  | 0.039 | 0.019-0.066 | 0.65 |
| ≥56 | 7 |  | <0.01 | 68% |  | random |  | 0.047 | 0.012-0.096 |  |
| Gender (Male%) |  |  |  |  |  |  |  |  |  |  |
| <50% | 7 |  | <0.01 | 70% |  | random |  | 0.023 | 0.006-0.048 | 0.21 |
| ≥50% | 4 |  | <0.01 | 78% |  | random |  | 0.066 | 0.006-0.167 |  |
| Previous first line systemic chemotherapy (%) |  |  |  |  |  |  |  |  |  |  |
| <100% | 7 |  | <0.01 | 81% |  | random |  | 0.024 | 0.002-0.062 | 0.22 |
| 100% | 4 |  | 0.77 | 0% |  | common |  | 0.049 | 0.026-0.077 |  |
| Concomitant systemic chemotherapy (%) |  |  |  |  |  |  |  |  |  |  |
| <100% | 6 |  | <0.01 | 67% |  | random |  | 0.041 | 0.010-0.087 | 0.99 |
| 100% | 4 |  | 0.62 | 0% |  | common |  | 0.041 | 0.020-0.066 |  |
| PIPAC procedure more than 3 times (%) |  |  |  |  |  |  |  |  |  |  |
| <30% | 5 |  | 0.23 | 29% |  | common |  | 0.033 | 0.013-0.060 | 0.42 |
| ≥30% | 6 |  | 0.04 | 58% |  | random |  | 0.049 | 0.018-0.091 |  |
| Study type |  |  |  |  |  |  |  |  |  |  |
| Retrospective | 7 |  | <0.01 | 83% |  | random |  | 0.045 | 0.010-0.097 | 0.27 |
| Prospective | 5 |  | 0.39 | 3% |  | common |  | 0.023 | 0.010-0.040 |  |
| **PIPAC-related mortality (CTCAE 5)** |  |  |  |  |  |  |  |  |  |  |
| PCI |  |  |  |  |  |  |  |  |  |  |
| <18 | 8 |  | 0.46 | 0% |  | common |  | 0.002 | 0.000-0.009 | 0.55 |
| ≥18 | 2 |  | 0.82 | 0% |  | common |  | 0.000 | 0.000-0.013 |  |
| Age |  |  |  |  |  |  |  |  |  |  |
| <56 | 4 |  | 0.71 | 0% |  | common |  | 0.003 | 0.000-0.016 | 0.92 |
| ≥56 | 7 |  | 0.84 | 0% |  | common |  | 0.001 | 0.000-0.008 |  |
| Gender (Male%) |  |  |  |  |  |  |  |  |  |  |
| <50% | 7 |  | 0.78 | 0% |  | common |  | 0.001 | 0.000-0.007 | 0.50 |
| ≥50% | 4 |  | 0.32 | 14% |  | common |  | 0.003 | 0.000-0.018 |  |
| Previous first line systemic chemotherapy (%) |  |  |  |  |  |  |  |  |  |  |
| <100% | 7 |  | 0.46 | 0% |  | common |  | 0.001 | 0.000-0.007 | 0.63 |
| 100% | 4 |  | 0.74 | 0% |  | common |  | 0.002 | 0.000-0.014 |  |
| Concomitant systemic chemotherapy (%) |  |  |  |  |  |  |  |  |  |  |
| <100% | 6 |  | 0.67 | 0% |  | common |  | 0.003 | 0.000-0.012 | 0.89 |
| 100% | 4 |  | 0.72 | 0% |  | common |  | 0.002 | 0.000-0.013 |  |
| PIPAC procedure more than 3 times (%) |  |  |  |  |  |  |  |  |  |  |
| <30% | 5 |  | 0.70 | 0% |  | common |  | 0.000 | 0.000-0.007 | 0.22 |
| ≥30% | 6 |  | 1.00 | 0% |  | common |  | 0.003 | 0.000-0.012 |  |
| Study type |  |  |  |  |  |  |  |  |  |  |
| Retrospective | 7 |  | 0.32 | 15% |  | common |  | 0.000 | 0.000-0.006 | 0.96 |
| Prospective | 5 |  | 0.99 | 0% |  | common |  | 0.001 | 0.000-0.009 |  |
| **1-year of overall survival** |  |  |  |  |  |  |  |  |  |  |
| PCI |  |  |  |  |  |  |  |  |  |  |
| <18 | 7 |  | <0.01 | 69% |  | random |  | 0.561 | 0.463-0.656 | 0.86 |
| ≥18 | 3 |  | 0.58 | 0% |  | common |  | 0.576 | 0.452-0.696 |  |
| Age (years) |  |  |  |  |  |  |  |  |  |  |
| <56 | 3 |  | 0.09 | 59% |  | random |  | 0.606 | 0.452-0.751 | 0.13 |
| ≥56 | 5 |  | 0.19 | 32% |  | common |  | 0.466 | 0.407-0.525 |  |
| Gender (Male%) |  |  |  |  |  |  |  |  |  |  |
| <50% | 7 |  | <0.01 | 67% |  | random |  | 0.578 | 0.486-0.668 | 0.19 |
| ≥50% | 4 |  | 0.09 | 55% |  | random |  | 0.460 | 0.315-0.609 |  |
| Previous first line systemic chemotherapy (%) |  |  |  |  |  |  |  |  |  |  |
| <100% | 7 |  | 0.04 | 54% |  | random |  | 0.528 | 0.444-0.611 | **0.02** |
| 100% | 3 |  | 0.48 | 0% |  | common |  | 0.690 | 0.581-0.790 |  |
| Concomitant systemic chemotherapy (%) |  |  |  |  |  |  |  |  |  |  |
| <100% | 5 |  | 0.93 | 0% |  | common |  | 0.470 | 0.406-0.535 | **0.01** |
| 100% | 3 |  | 0.25 | 28% |  | common |  | 0.659 | 0.555-0.756 |  |
| PIPAC procedure more than 3 times (%) |  |  |  |  |  |  |  |  |  |  |
| <30% | 3 |  | 0.64 | 0% |  | common |  | 0.494 | 0.385-0.604 | 0.53 |
| ≥30% | 4 |  | 0.01 | 71% |  | random |  | 0.552 | 0.411-0.690 |  |
| Study type |  |  |  |  |  |  |  |  |  |  |
| Retrospective | 5 |  | 0.23 | 29% |  | common |  | 0.652 | 0.584-0.718 | **0.01** |
| Prospective | 4 |  | 0.79 | 0% |  | common |  | 0.470 | 0.402-0.538 |  |
| **Median overall survival** |  |  |  |  |  |  |  |  |  |  |
| PCI |  |  |  |  |  |  |  |  |  |  |
| <18 | 6 |  | <0.01 | 94% |  | random |  | 12.19 | 8.94-15.43 | 0.40 |
| ≥18 | 3 |  | <0.01 | 84% |  | random |  | 9.82 | 5.37-15.26 |  |
| Age (years) |  |  |  |  |  |  |  |  |  |  |
| <56 | 5 |  | <0.01 | 97% |  | random |  | 11.59 | 7.32-15.86 | 0.92 |
| ≥56 | 4 |  | <0.01 | 79% |  | random |  | 11.34 | 8.46-14.21 |  |
| Gender (Male%) |  |  |  |  |  |  |  |  |  |  |
| <50% | 6 |  | <0.01 | 90% |  | random |  | 12.98 | 10.22-15.75 | **0.01** |
| ≥50% | 3 |  | 0.42 | 0% |  | common |  | 7.43 | 6.34-8.52 |  |
| Previous first line systemic chemotherapy (%) |  |  |  |  |  |  |  |  |  |  |
| <100% | 5 |  | <0.01 | 76% |  | random |  | 11.76 | 9.90-13.62 | 0.71 |
| 100% | 4 |  | <0.01 | 93% |  | random |  | 12.86 | 7.42-18.30 |  |
| Concomitant systemic chemotherapy (%) |  |  |  |  |  |  |  |  |  |  |
| <100% | 5 |  | <0.01 | 78% |  | random |  | 10.85 | 8.50-13.21 | 0.09 |
| 100% | 4 |  | <0.01 | 91% |  | random |  | 14.49 | 11.01-17.98 |  |
| PIPAC procedure more than 3 times (%) |  |  |  |  |  |  |  |  |  |  |
| <30% | 5 |  | <0.01 | 87% |  | random |  | 9.51 | 6.93-12.09 | **0.02** |
| ≥30% | 4 |  | <0.01 | 92% |  | random |  | 14.21 | 11.34-17.08 |  |
| Study type |  |  |  |  |  |  |  |  |  |  |
| Retrospective | 5 |  | <0.01 | 97% |  | random |  | 12.57 | 8.35-16.78 | 0.15 |
| Prospective | 5 |  | <0.01 | 81% |  | random |  | 10.99 | 8.61-13.38 |  |
