# Supplementary figures and images for: Pressurized intraperitoneal aerosol chemotherapy in advanced gastric cancer with peritoneal metastases: a comprehensive meta-analysis of feasibility, efficacy, and safety
Source: Gastroenterol Rep (Oxf). 2025 Jun 15;13:goaf040. doi: 10.1093/gastro/goaf040 (PMC12167634; doi:10.1093/gastro/goaf040)

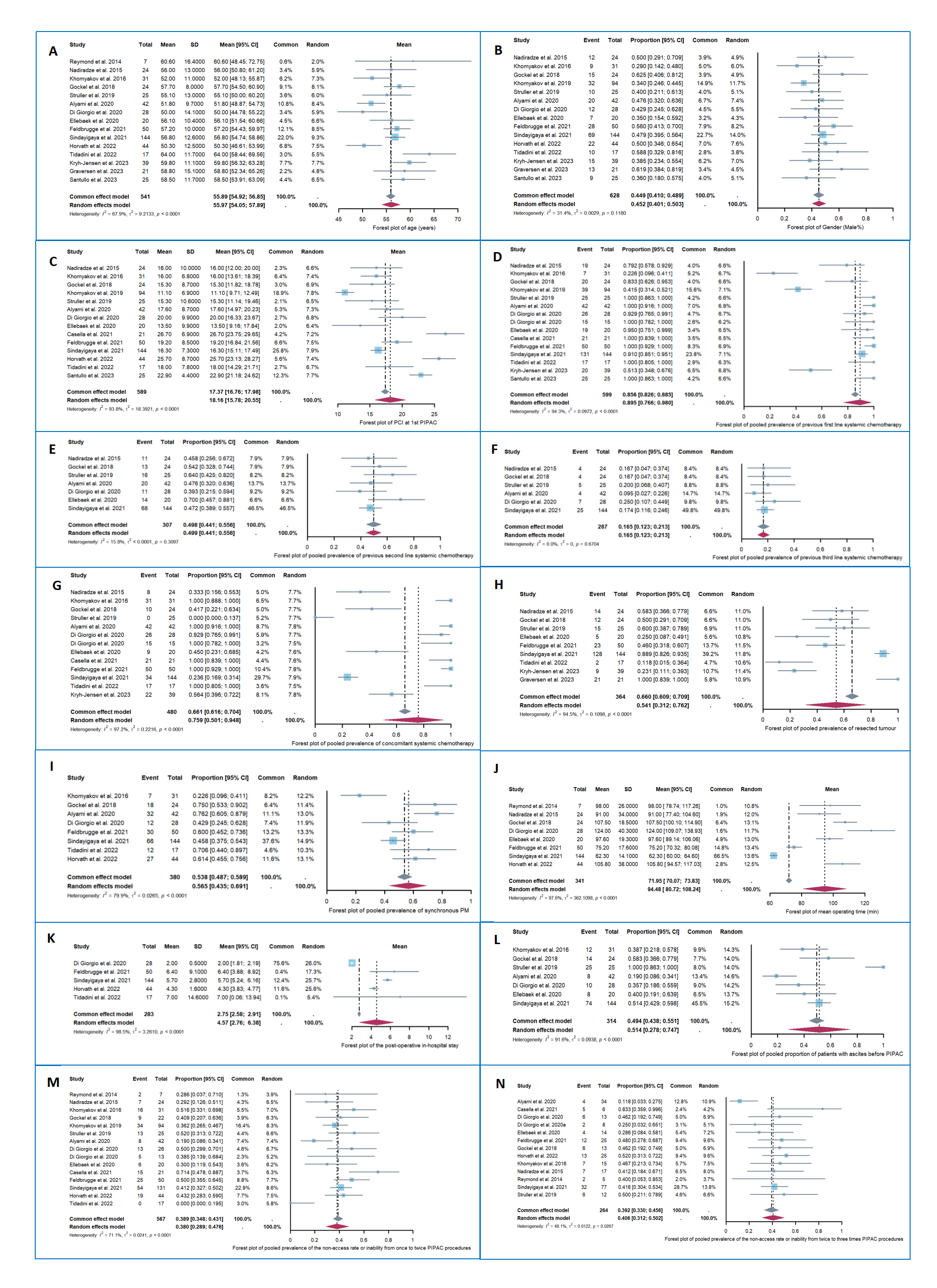

Supplement: goaf040_Supplementary_Data [file goaf040_supplementary_data.zip › 2024-231 Supplementary Figure S1.tiff]

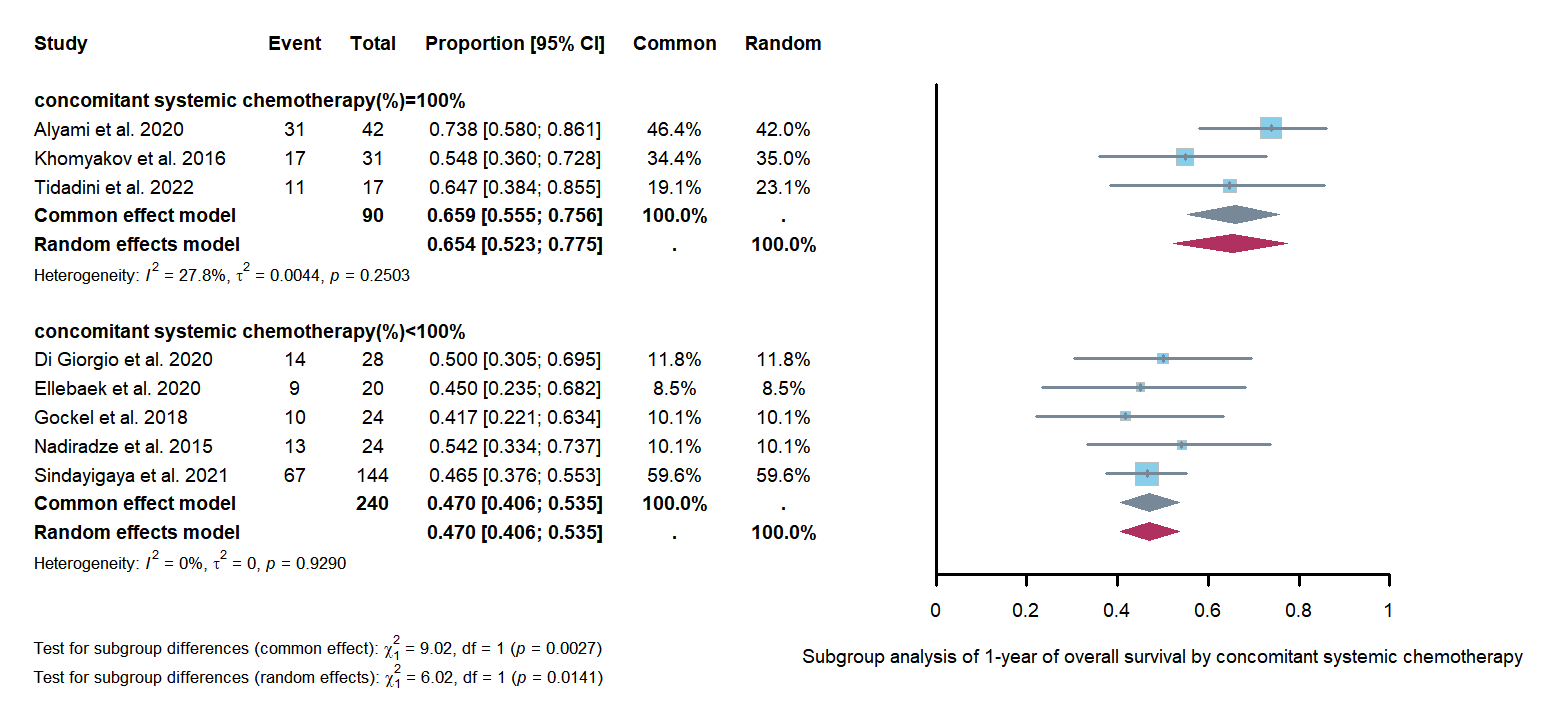

Supplement: goaf040_Supplementary_Data [file goaf040_supplementary_data.zip › 2024-231 Supplementary Figure S2.tiff]

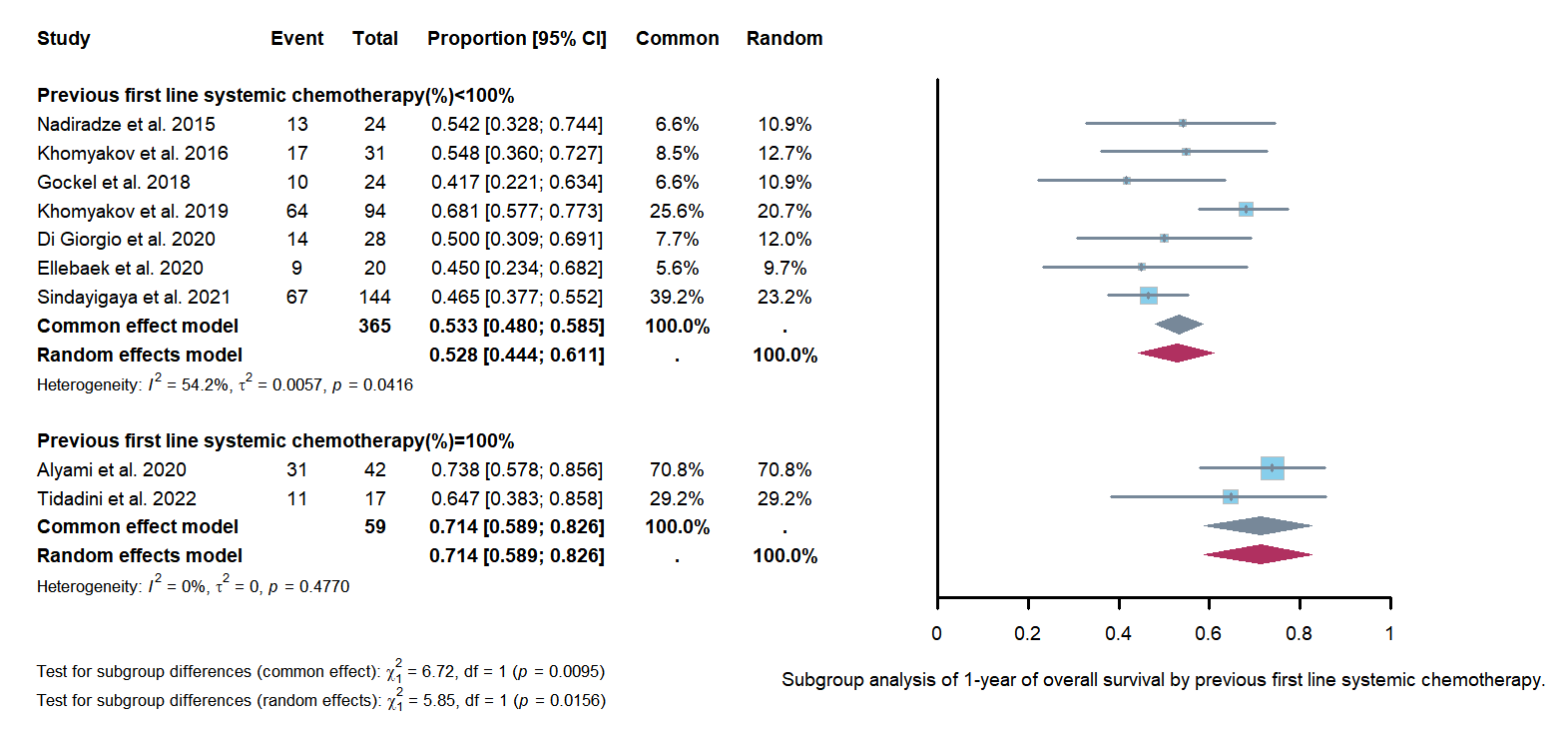

Supplement: goaf040_Supplementary_Data [file goaf040_supplementary_data.zip › 2024-231 Supplementary Figure S3.tiff]

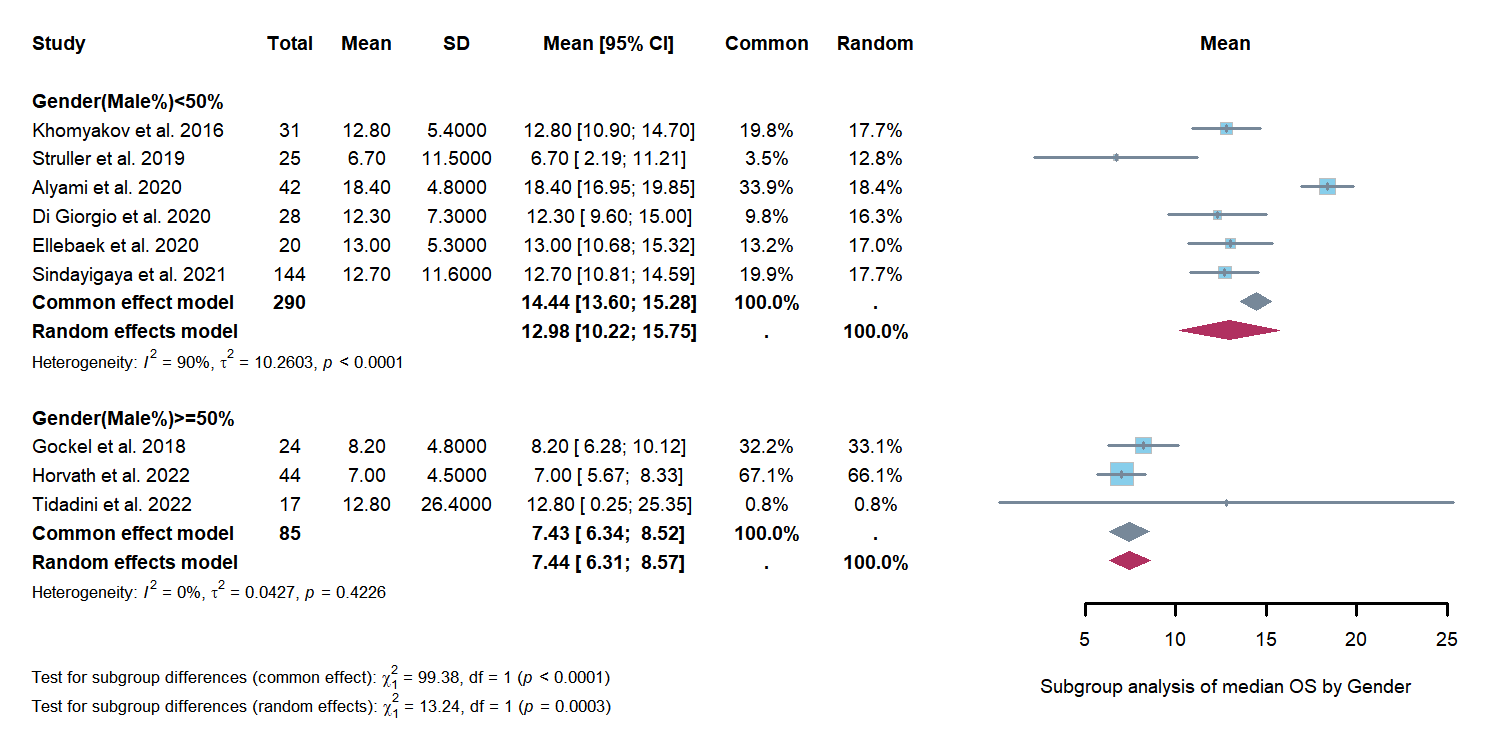

Supplement: goaf040_Supplementary_Data [file goaf040_supplementary_data.zip › 2024-231 Supplementary Figure S4.tiff]

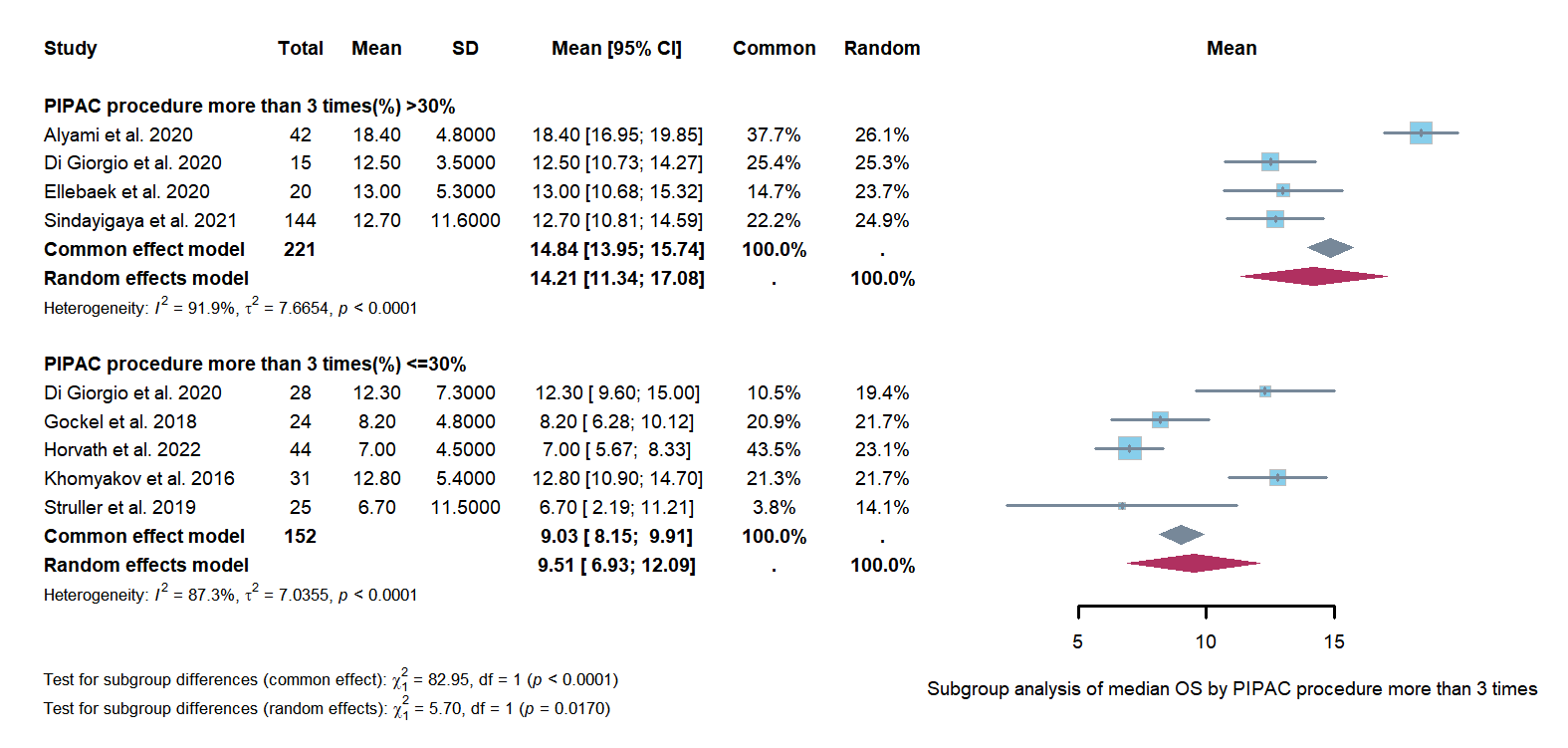

Supplement: goaf040_Supplementary_Data [file goaf040_supplementary_data.zip › 2024-231 Supplementary Figure S5.tiff]
